# Chemical and Biological Investigations of *Allium scorodoprasum* L. Flower Extracts

**DOI:** 10.3390/ph16010021

**Published:** 2022-12-23

**Authors:** Nikoleta Đorđevski, Abdullahi Ibrahim Uba, Gokhan Zengin, Jelena Božunović, Uroš Gašić, Elizabeta Ristanović, Ana Ćirić, Biljana Nikolić, Dejan Stojković

**Affiliations:** 1 Laboratory of Immunology, Institute of Microbiology, Medical Military Academy, Crnotravska 17, 11000 Belgrade, Serbia; 2 Department of Molecular Biology and Genetics, Faculty of Engineering and Natural Sciences, Kadir Has University, 34083 Istanbul, Turkey; 3 Physiology and Biochemistry Research Laboratory, Department of Biology, Science Faculty, Selcuk University, 42130 Konya, Turkey; 4 Department of Plant Physiology, Institute for Biological Research “Siniša Stanković”—National Institute of Republic of Serbia, University of Belgrade, Bulevar Despota Stefana 142, 11000 Belgrade, Serbia; 5 University of Belgrade, Faculty of Biology, Studentski trg 16, 11000 Belgrade, Serbia

**Keywords:** *Alllium scorodoprasum* L. flowers, anti-enzymatic, antimicrobial, antioxidant, clinical isolates, medieval remedy, molecular modeling, wound healing

## Abstract

This study was designed to investigate the impact of different extraction solvent systems on the chemical composition and biological activities of *Allium scorodoprasum* L. (Amaryllidaceae)—the medicinal plant that was traditionally used as a remedy in the medieval period in the Balkans. Targeted chemical analysis of nine different extracts was performed by UHPLC(−)HESI–QqQ-MS/MS. Antimicrobial and antibiofilm activities of the extracts were investigated on sixteen clinical isolates of bacteria, yeasts and dermatomycetes, all isolated from infected human skin and corneal formations. Cytotoxicity and wound-healing properties were tested on human immortalized keratinocytes (HaCaT cell line). Antioxidant activity was assessed by six different assays, while beneficial potential against certain neurodegenerative diseases and type 2 diabetes was determined in selected enzyme inhibition assays coupled with molecular modeling. The results showed that the obtained extracts were rich in phenolic compounds, especially flavonoid glycosides such as rutin and kaempferol 3-*O*-glucoside. All of the extracts showed antimicrobial, wound-healing, antioxidant and anti-enzymatic properties. This study is the first of its kind, linking the medieval medicinal use of wild-growing flowers of *A. scorodoprasum* with contemporary in vitro scientific approaches.

## 1. Introduction

The Hilandar medical codex represents a unique South Slavic anthology of medieval medicine [1,2]. It contains remedial/healing compositions that were used within the medieval Balkan countries that have not been preserved in other South Slavic manuscripts. According to paleographical analysis, it was written in the second half of the 15th and the first half of the 16th century [1,2,3]. Specifically, the Hilandar medical codex describes the use of dried flowers collected from wild *Allium* spp. in treating skin wounds and infections, applied in the form of powdered flowers.

The genus *Allium* belongs to the family Amaryllidaceae, chiefly distributed in the tropics and sub-tropics, including the Mediterranean coast, Africa, Asia and South America; these plants are highly adapted and have a high level of speciation [4,5]. *Allium* species are distributed in moderate, arid, semi-arid and subtropical areas such as the Mediterranean, Central Asia, Africa and parts of Europe. As herbaceous geophilic perennials, *Allium* includes many species with sharp linear leaves that could arise from a bulb or rhizome [6,7]. The genus *Allium* represents one of the largest genera of monocotyledons, which includes approximately 915 species, including culinary herbs such as garlic, onion, shallot, leek, chive and spring onion [8]. *A. scorodoprasum* L. is a 25–90-cm-tall plant, with bulbs 1–2 cm in diameter. It has leaves of 2–5 threads, within 2–8 mm, while the flowers are dark red and purple. It grows in limestone areas and rural areas, grasslands and clayey slopes [9].

Traditional medicine indicates that regular consumption of herbs belonging to the genus *Allium* is beneficial for human health, at least due to the rich content of antioxidant compounds. *Allium* species are among the oldest cultivated plants in the world [10]. *A. scorodoprasum*, snake onion, also known as wild leek, is an annual plant whose leaves and bulbs can be consumed raw or cooked, as well as a commonly used spice in the production of cheese, yogurt and bread [11]. In addition, it is used not only as a culinary herb, but also as a folk medicine, i.e., as an antiseptic for wound healing, as a hypertension treatment and as a diuretic; additionally, it can prevent aging and cardiovascular and liver diseases, treat diabetes and improve eyesight [9].

The main bioactive compounds of *Allium* spp. include organic sulfur compounds (OSCs) and polyphenols [12]. However, due to the complex chemistry of OSCs, because of their highly volatile and thermally unstable nature, as well as their complex structure contributing to poor digestibility, their health benefits are most likely reduced and/or hardly utilized [12,13,14,15]. Polyphenols, which are much more stable than OSCs and have antioxidant properties, dominate, as more sustainable bioactive ingredients, in *Allium* species.

Type 2 diabetes and Alzheimer’s disease (AD) are among the main global health problems today. Approximately 422 million people worldwide are affected by type 2 diabetes and contribute to a significant economic loss (WHO, 2017). There is increasing evidence that points to a connection between AD and type 2 diabetes. AD is the most common form of dementia and affects more than 35 million people worldwide [16]. In addition, these two diseases have a common causative factor—oxidative stress [17]. Chronic diabetes is characterized by the decreased self-healing ability of the skin. Because of this, even the smallest wounds on the feet can lead to the appearance of ulcers—diabetic ulcers. These are chronic wounds that are difficult to heal or even completely incurable, representing suitable grounds for the development of various infections. Such infections can lead to amputation of the lower extremities, and in some cases even death [18]. Diabetes leads to disorders of leukocyte function, as well as to decreased chemotaxis and consequent inadequate migration of neutrophils and macrophages into the wound [19,20]. An increased risk of wound and skin infections is seen not only in diabetic patients, but also in the general population. Indeed, skin and soft tissue infections (SSTIs) represent one of the most common forms of infection today [21]. This trend has occurred due to the increasing size of the elderly population and immunocompromised patients, as well as the increasing resistance of pathogens to existing antimicrobial agents [22].

Accordingly, the aim of the present study was to investigate the potential of different solvents to extract biologically active substances from *A. scorodoprasum* flowers, previously described in South Slavic medieval medical books as a therapeutic for skin issues, and to estimate the biological activity of the obtained extracts. Furthermore, the chemical composition of the extracts was investigated, as well as their antimicrobial and antibiofilm activities against clinical bacterial and fungal isolates obtained from patients with SSTIs. Antioxidant, anti-enzymatic (antidiabetic and anti-neurodegenerative) and wound-healing properties were also investigated in vitro.

## 2. Results and Discussion

A total of nine extracts were used for the experiments. The general idea was to investigate the influence of different extraction solvents on *A. scorodoprasum* flowers’ chemical composition and biological activities connected with their traditional medieval use. The yields of the extracts E10, EW7.5, EW5, EW2.5, W10, B10, BE7.5, BE5 and BE2.5 are presented in Table 1. The influence of different extraction solvents on biological activity and chemical composition was previously reported for other plant species [23].

### 2.1. Chemical Composition of A. scorodoprasum Flower Extracts as Influenced by the Extraction Solvents

We have detected a range of polyphenolic molecules in *A. scorodoprasum* flower extracts, with the EW5 extract being the most abundant in identified polyphenolic compounds (12,461.22 mg/kg), followed by the E10 extract (7610.35 mg/kg) (Table 2). Phenolic acids, flavonoid aglycones and glycosides were found in the investigated extracts; flavonoids dominated over phenolic acids. Generally, the most dominant compounds found in the extracts were flavonoids, e.g., rutin, kaempferol, kaempferol 3-*O*-glucoside and isorhamnetin, with rutin being the most abundant in E10, EW7.5, EW5 and BE5; kaempferol 3-*O*-glucoside dominating in EW2.5, B10 and BE2.5; and kaempferol in BE7.5, while isorhamnetin was found as the most dominant compound in the extract W10. All of the dominant compounds mentioned were found in all of the investigated extracts. It could be noted that the polarity of the solvent system influenced the yield of extracted compounds. Namely, solvent systems with a high share of ethanol (extracts E10, EW7.5, EW5) were found to be the best for the extraction of high rutin concentrations from the investigated material of *A. scorodoprasum*. According to our knowledge, this is the first study investigating the influence of extraction solvents on the chemical composition of *A. scorodoprasum* flowers. Previous studies on *Allium ursinum* flowers also showed that the water:methanol (50:50) extract of the flowers contained rutin (19.7 mg/100 g) [24]. *p*-Coumaric acid (981.9, 952.6 μg/g extract) was the major compound of the flowers of *Allium paniculatum* subsp. *villosulum* and *Allium paniculatum* subsp. *paniculatum* [25]. This phenolic acid was also recorded in our study, but it was not dominant in the flower extracts of *A. scorodoprasum*. Previous chemical analysis of the methanolic extract obtained from *A. scorodoprasum* subsp. *rotundum* flowers from Turkey indicated that the major compound in the extract was rosmarinic acid [26], and in contrast to our study, phenolic acids dominated over flavonoids. These differences in the chemical composition of *A. scorodoprasum* could be attributed to the different environmental conditions in Serbia and Turkey, the sampling period of plants and different extraction solvents used.

### 2.2. Identification of Microbes and Antimicrobial Activity

MALDI-TOF/MS identification of the isolates collected from infected human skin and corneal formations revealed the presence of the following bacteria: *Staphylococcus epidermidis*, *Staphylococcus lugdunensis* and *Proteus vulgaris*, yeasts: *Candida albicans*, *Candida krusei*, *Candida tropicalis* and *Candida kefyr* and dermatomycetes: two isolates of *Microsporum fulvum*, two isolates of *Microsporum canis*, two isolates of *Trichophyton rubrum*, two isolates of *Trichophyton mentagrophytes* and one isolate of *Trichophyton violaceum* (Table 3). Our results are in accordance with previous findings reporting the microbial landscape of healthy skin and skin lesions [27]. The most commonly isolated bacteria from the skin and skin lesions included the following: Actinobacteria (*Corynebacterium* spp.; *Microbacterium* spp.; *Micrococcus* spp.), Firmicutes non-haemolytic facultative anaerobic staphylococci (*Staphylococcus* spp.), α-hemolytic streptococci (*Streptococcus* spp.), enterococci (*Enterococcus* spp.), Bacteroidetes (*Sphingobacterium* spp., *Chryseobacterium* spp.), Proteobacteria (*Janthinobacterium* spp., *Serratia* spp., *Halomonas* spp., *Delftia* spp., *Comamonas* spp.). The skin may also be colonized by pathogenic bacteria, e.g., group A streptococci (*S. pyogenes*), golden *Staphylococcus* (*S. aureus*) and Gram (-) bacilli (*Pseudomonas aeruginosa*), and also by aerobic coryneform bacteria (*Corynebacterium* spp.), which can cause an infection as well. Among others, on human skin, the following fungi were previously reported: *Penicillium* spp., *Aspergillus* spp., *Candida* spp., *Chaetomium* spp., *Chrysosporium* spp., *Cladosporium* spp., *Mucor* spp., *Debaryomyces* spp., *Cryptococcus* spp., *Microsporum* spp., *Epidermophyton* spp., *Trichophyton* spp. [27,28,29].

All of the isolated and identified microorganisms were used for further antimicrobial assays. The extracts showed very good effects against all tested bacteria and yeasts (Table 4). Extracts E10, BE7.5, BE5 and BE2.5 showed the best effect on *S. epidermidis*, with an MIC 0.5 mg/mL. Extracts EW7.5 and EW5 showed the most prominent effect against both treated bacteria from the genus *Staphylococcus*, with an MIC of 0.5 mg/mL, while extract EW2.5 showed the strongest activity against *S. lugdunensis*. Extract W10 showed the best effect against *S. epidermidis* and *P. vulgaris*, with an MIC of 0.5 mg/mL. *P. vulgaris* could be denoted as the most sensitive bacterium. Concerning the sensitivity of staphylococci, *S. epidermidis* was more susceptible than *S. lugdunensis*. Further, comparing the relative efficiency of the extracts, we found the **B10** extract to be the one with the highest antibacterial activity (at least for *P. vulgaris* and *S. epidermidis*) and the BE2.5 extract as the one with the weakest antibacterial effect (at least for *P. vulgaris* and *S. lugdunensis*). Taking into account both the chemical composition (Table 2) and antibacterial activity (Table 3), one could note that the dominant constituents of extract B10—namely flavonol glucosides such as kaempferol 3-*O*-glucoside (being highly contained actually in this extract, with 2348.39 mg/kg dw) and rutin (1936.22 mg/kg dw) and additionally flavonol kaempferol (1407.73 mg/kg dw)—could be responsible for the observed antimicrobial activity. Literature data indicating their antibacterial activity [30,31,32,33] are in line with this result. Certainly, interactions of bioactive constituents and a possible synergism could not be excluded. 

Extract E10 showed the best effect against the yeast *C. albicans*, with an MIC of 0.06 mg/mL (Table 4). Extract EW7.5 showed the weakest effect against *C. krusei*, with an MIC of 2 mg/mL, while it showed the same effect against the other three yeasts, with MICs of 0.125 mg/mL. EW5 extract showed the best effect against *C. albicans* and *C. tropicalis*, with an MIC of 0.06 mg/mL. The EW2.5 extract had the best effect on the yeast *C. albicans*, with an MIC of 0.125 mg/mL. The extract W10 showed its strongest effect against the yeast *C. albicans*, with an MIC of 0.25 mg/mL. Extracts B10, BE7.5 and BE5 showed equal activity against all yeasts, with MICs of 0.5 mg/mL. The BE2.5 extract showed its best effect against *C. albicans*, with an MIC of 0.5 mg/mL. Comparing all obtained MIC values among tested yeast species revealed that *C. albicans* could be denoted as the most sensitive strain, especially in the case of E10 and EW5, with a determined MIC of 0.06 mg/mL. In addition, EW5 also showed a notable anticandidal effect against *C. tropicalis*, with an MIC of 0.06 mg/mL. Indeed, **EW5** could be determined as the most efficient anticandidal agent, while the extract with the weakest anticandidal effect was BE2.5 (Table 4). 

*A. scorodoprasum* flower extracts showed some lower but still notable antifungal activity against selected strains of dermatomycetes (Table 5). Extract E10 showed the best effect on both isolates of the species *T. rubrum* and *M. canis* and additionally on one isolate of *M. fulvum*, with MIC 1 mg/mL; the same was true for extract E7.5, with the exception of *M. fulvum*, where the MIC for this extract was 2 mg/mL. Extracts E5 and E2.5 showed the best effect on *T. rubrum*, with an MIC of 0.25 mg/mL, followed by *M. canis* (MIC 0.5 mg/mL). Extract W10 showed equal effects on *T. rubrum* and *M. canis*, with MIC 2 mg/mL, and with MIC 4 mg/mL for all other treated fungi. All the extracts with butanol showed the same weaker effect against all tested fungi, with MIC 4 mg/mL. 

Comparing all MIC values determined on dermatomycetes pointed out that the most sensitive dermatomycete was *T. rubrum*, followed by *M. canis*, at least for the ethanolic and water extracts (E10, EW7.5, EW5, EW2.5 and W10). On the contrary, the most susceptible dermatomycetes could not be determined for butanol-containing extracts (B10, BE7.5, BE5 and BE2.5), since all of them induced equal and relatively weak effects. Furthermore, the extract **EW5** was determined as the most prosperous one. 

Taking together the activity against both candidal and dermatomycetal isolates, fungi were more sensitive to ethanol:water extracts than to butanol:ethanol extracts, with the exception of *C. krusei*. Furthermore, if the polyphenolic profile of the extracts was taken into consideration, the obtained results indicated that the antifungal activity could be related to the overall polyphenolic content, since the **EW5** extract contained the highest amount of polyphenolic compounds, especially flavonoids, and was shown to be the best antifungal agent. Indeed, among the determined polyphenols, some flavonoids have already been identified as antifungal agents influencing the expression of various virulence factors and consequently fungal pathogenicity. Rutin was previously shown to possess antifungal activity at different concentrations ranging from 40 µg/mL to >1000 µg/mL [34]. 

Further, the comparison of all determined antimicrobial effects indicated that the general susceptibility of the isolates to the tested extracts could be given in the following order: the most sensitive were isolates of *Candida* sp., followed by bacterial species and finally dermatomycetes. While ethanol-containing and water extracts were more efficient against fungi, butanol-containing extracts, especially B10, had a better effect on the growth inhibition of bacteria. The difference in the antibacterial and antifungal activity of the extracts could be attributed to the differences between the prokaryotic nature of bacteria and the eukaryotic nature of fungi. It seems that the compounds in the extracts acted synergistically; however, rutin was probably the most responsible for the antifungal activity, while the specific balance in concentrations of kaempferol 3-*O*-glucoside and rutin in the B10 extract most likely had the best impact on the antibacterial activity.

Regarding the antibiofilm activity of *A. scorodoprasum* flower extracts, against the formation of *S. lugdunensis* biofilms, three extracts that exhibited the strongest effect on its planktonic growth, with an MIC of 0.5 mg/mL (EW7.5, EW5 and EW2.5), were chosen. Three concentrations of the extracts were used: MIC/2, MIC and MBC. All three extracts had a very good effect in preventing biofilm formation (Table 6). They had the highest percentages for the prevention of biofilm formation when applying MBC concentrations. The extract with the highest effect was **EW5**, with 70.59% inhibition of biofilm formation.

Concerning the dominant phenolics of these three biofilm-inhibitory extracts and their possible contributions to the observed antibiofilm effect, as far as we know, there are no available data on antibiofilm potential on *S. lugdunensis*. However, literature data revealed that, among others, rutin (the most dominant constituent of EW7.5 and EW5, and additionally one of the dominant constituents of EW2.5) has already been confirmed as an antibiofilm agent in other model bacteria, namely in *Pseudomonas aeruginosa* [32], *Klebsiella pneumonia* [33] and *Escherichia coli* and *Staphylococcus aureus* [35]. In addition, kaempferol 3-*O*-glucoside (astragalin), the constituent with the highest share in EW2.5, could also contribute to the observed antibiofilm activity. 

The same extracts (EW7.5, EW5 and EW2.5), but applied in different concentrations (MIC, MBC, 2xMBC), were also selected to screen the already formed biofilm eradication. As shown in Table 7, the extracts were not so successful in removing the already formed biofilm at the tested concentrations, except in the case of the **EW2.5** extract, which was successful in eradicating 46.6% of the biofilm at the 2xMBC concentration.

### 2.3. Cytotoxicity and Wound-Healing Properties of A. scorodoprasum

**Figure 1:**
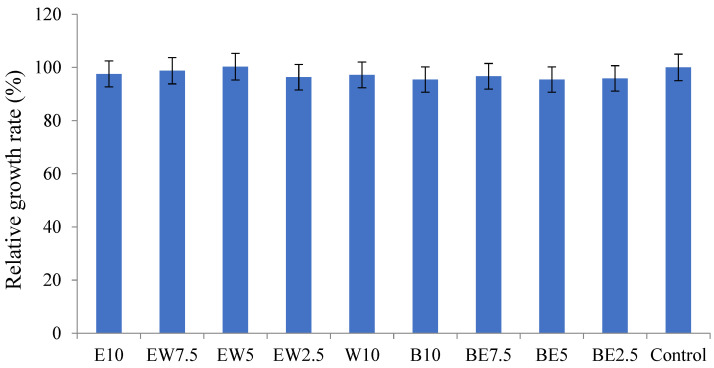
Relative growth rate of HaCaT cells in the presence of *A. scorodoprasum* flower extracts at concentration 400 μg/mL.

The effect of *A. scorodprasum* flower extracts on viability was tested in a HaCaT cell model. HaCaT cells are a human immortalized keratinocyte cell line that is frequently used in assays elucidating the safety of newly designed natural products [23,36]. It is evident from Figure 1 that all of the tested extracts of *A. scorodoprasum* did not induce significant changes in the viability of HaCaT cells when compared to the control non-treated cells. The extracts were tested at different concentrations ranging from 12.5 μg/mL to 400 μg/mL. According to the methodology developed, natural compounds exhibiting a cytotoxic effect with IC_50_ value ˃ 400 μg/mL are considered non-cytotoxic. This primary laboratory screening of cytotoxicity leads to the conclusion that *A. scorodoprasum* extracts are potentially safe for further applications. 

A timely wound-healing process is important in diabetic patients and the elderly suffering from chronic or acute wounds. Non-treated and open wounds are exposed to different microbial infections; therefore, natural products exhibiting antimicrobial and wound-healing capacities are of prime importance for patients. The extract that proved to be the best in the wound-healing process was **EW5**, with 100% healing capacity after 48 h of exposure, being followed by E10 (81%). These two extracts particularly stood out for their efficiency, while all others showed a slightly higher wound closure capacity than control non-treated cells (Table 8). These results indicate that *A. scorodoprasum* flowers are a good source of compounds that can have wound-healing potential, as described in medieval medical books. This is the first scientific confirmation of the use of wild *Allium spp*. flowers in the treatment of wounds, but some literature data confirm some of the dominant constituents as wound-healing agents. For example, rutin promoted wound healing in vivo, in hyperglycemic rats [37], while kaempferol was effective both in diabetic and nondiabetic rats [38]. Further, kaempferol and its derivative, kaempferol 3-*O*-glucoside, also showed a wound-healing capacity in the study in [39]. 

### 2.4. Antioxidant Properties of the Extracts 

The antioxidant activity of the extracts was evaluated with the following antioxidant assays: DPPH, ABTS, CUPRAC, FRAP, metal chelating and phosphomolybdenum (Table 9). The highest antioxidant capacity was shown for extract **EW5** in almost all of the assays except FRAP, according to which it had the second-highest activity among those measured, while the highest ferric reducing antioxidant power was found for the E10 extract. BE5 was singled out as the extract with the lowest antioxidant activity, with the lowest concentrations in all of the assays. A previous study investigated the antioxidant capacity of *A. scorodoprasum* ethanolic extract by three methods (FRAP, TEAC and DPPH), calculated as trolox equivalents [40]. The authors indicated that the FRAP, TEAC and DPPH values were calculated as 2.89, 2.72 and 1.63 μmol TE/g extract, respectively. These results are comparable to the ones obtained in our current study, although our study was limited to the antioxidant activity of *A. scorodoprasum* flower extracts. It was shown in a previous investigation that flower methanolic extracts obtained from *A. scorodoprasum* subsp. *rotundum* from Turkey had antioxidant properties, as evaluated by the same assays used in our study, i.e., DPPH, ABTS, CUPRAC, FRAP, metal chelating and phosphomolybdenum, and the results were expressed in the same units [26]. Although the authors investigated methanolic extracts from the flowers, the results were within the range of those obtained in our study. This is the first study exploring the antioxidant potential of different extracts from *A. scorodoprasum* growing wild in Serbia. 

### 2.5. Inhibitory Activity of Allium scorodoprasum Extracts on Enzymes Linked with the Treatment of Neurodegenerative Diseases and Type 2 Diabetes and Molecular Docking

Extracts E10, EW7.5, EW2.5 and B10 showed similar effects on AChE, with concentrations from 3.04 GALAE/g to 3.08 GALAE/g. Another group consisted of the EW5, W10 and BE7.5 extracts, which had concentrations ranging from 2.83 GALAE/g to 2.99 GALAE/g in AchE (Table 10). The other two extracts, BE5 and BE2.5, were not active towards AChE. Further, the extracts E10, B10, BE7.5, BE5 and BE2.5 showed the highest inhibitory effects towards BChE, with concentrations ranging from 3.34 GALAE/g to 3.99 GALAE/g. Other extracts showed weaker effects, with concentrations from 1.81 GALAE/g to 2.61 GALAE/g. The extract that had the greatest effect on the AChE enzyme was B10, while the BE2.5 extract had the greatest effect on BChE. Since inhibitors of these two enzymes have an effect on neurodegenerative changes, such as those seen in Alzheimer’s disease, we can conclude that the most potent against both enzymes was **E10**, which could be highlighted as the most prosperous candidate that could prevent/diminish neurodegeneration in Alzheimer’s disorder.

As with the previous two enzymes, the inhibitors of enzyme tyrosinase also have an effect on some neurodegenerative conditions, such as Parkinson’s disease. The tested extracts were potent against this enzyme. Extracts W10 and EW5 had the weakest effect, with the lowest concentrations of 41.23 mg KAE/g and 43.96 mg KAE/g, respectively. The other extracts, except B10, had almost uniform potency ranging from 50.74 mg KAE/g to 65.34 mg KAE/g. The highest effect on the enzyme tyrosinase was achieved by extract **B10**, with the concentration of 75.66 mg KAE/g.

The inhibitors of the remaining two enzymes, amylase and glucosidase, play a role in type 2 diabetes therapy. The extracts had a fairly uniform effect against the α-amylase enzyme. The least effective was the EW2.5 extract, with the concentration of 0.10 mmol ACAE/g. The activities of the other extracts ranged from 0.19 mmol ACAE/g to 0.28 mmol ACAE/g. The extracts with the highest inhibitory activity were **E10** and **B10**. The extracts also showed a fairly equal effect, with concentrations from 0.86 mmol ACAE/g to 0.90 mmol ACAE/g towards the enzyme glucosidase. Only extract W10 was not active against this enzyme, while the extracts with the most prominent effects were EW7.5, EW2.5 and B10. We can conclude that the most potent extract for both of these enzymes was **B10**, with the highest concentrations for both extracts: 0.28 mmol ACAE/g for amylase and 0.90 mmol ACAE/g for glucosidase. It can be recommended to alleviate the symptoms of diabetes type 2. 

Previous investigations of *A. scorodoprasum* subsp. *rotundum* flower methanolic extracts also demonstrated their anti-enzymatic potential [26]. The methanolic extract showed AChE inhibition at 1.98 GALAE/g, while extracts in our current study had more pronounced effects, being up to 3.08 GALAE/g for the butanolic B10 extract [26]. Moreover, the methanolic extract was tested for BChE inhibition, with a result of 3.16 GALAE/g, while butanol–ethanol extract B2.5 in our study, tested for BChE inhibition, had a higher effect at 3.99 GALAE/g [26]. The extract that had the best inhibition effect towards tyrosinase in our study, with the value of 75.66 KAE/g, was the butanolic B10 extract, while the methanolic extract from the Mollica et al. [26] study had lower value of 55.21 KAE/g. These differences in the anti-enzymatic activity observed in our current study and the study by Mollica et al. [26] could be attributed to the different extraction solvents used and the chemical compounds identified in the samples. This is the first study investigating the impact of different extraction solvents on the enzyme-inhibitory activity of *A. scorodoprasum* flowers collected in Serbia.

**Figure 2:**
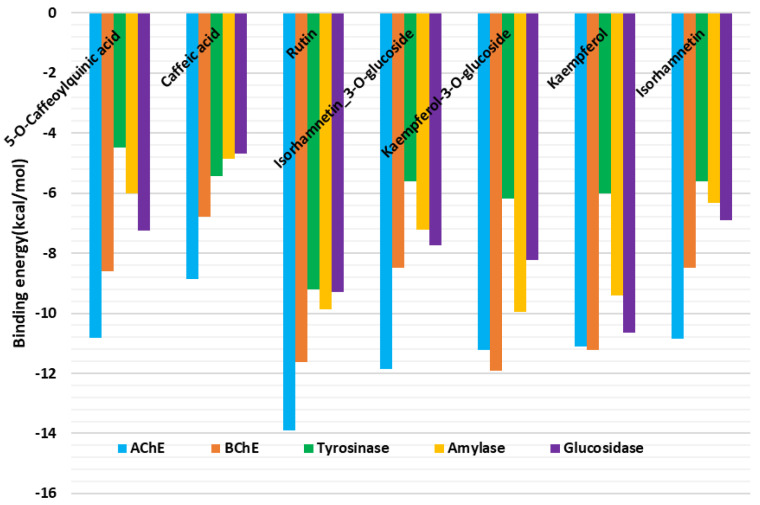
Binding energy (docking) scores of the bioactive compounds from the tested extracts against each of the target enzymes.

Molecular docking was performed to gain insights into the binding mode of the bioactive compounds and their interactions with the target enzymes. The binding energy (docking) score of each ligand against each enzyme is shown in Figure 2. 

**Figure 3:**
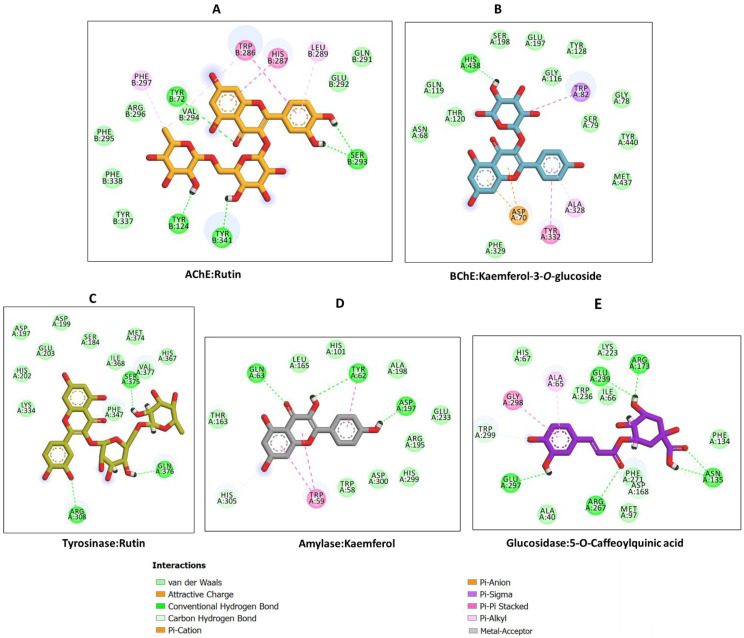
Protein–ligand interactions: (**A**) AChE and rutin, (**B**) BChE and kaempferol 3-*O*-glucoside, (**C**) tyrosinase and rutin, (**D**) amylase and kaempferol and (**E**) glucosidase and 5-*O*-caffeoylquinic acid.

Rutin was found to show a preference for AChE and bound to the rest of the target enzymes tightly. Kaempferol and kaempferol-3-*O*-glucosidase demonstrated high binding potential to BChE; they bound to amylase and glucosidase with high affinity and modestly to tyrosine. Hence, the protein–ligand interactions in some selected complexes were analyzed. H-bonds, several van der Waals interactions and a couple of hydrophobic and π–π stacked interactions are the major contributors to the interaction between some selected compounds (rutin, kaempferol-3-*O*-glucosidase, kaempferol and 5-*O*-caffeoylquinic acid) and AChE, BChE, amylase and glucosidase enzymes, respectively. Specifically, the binding of rutin and kaempferol 3-*O*-glucoside to AChE (Figure 3A) and BChE (Figure 3B) showed that these enzymes have relatively larger pockets that could accommodate compounds of different molecular sizes. Moreover, the presence of both polar and non-polar amino acid residues lining the enzymes’ channels allowed for the formation of the above-mentioned interactions, resulting in strong binding. On the other hand, tyrosinase, having a relatively narrow pocket, accommodated rutin, which was bound in the opposite direction to its orientation in the catalytic site of AChE, forming a couple of H-bonds and van der Waals interactions all over the channel (Figure 3C). Interestingly, kaempferol, with a relatively smaller size, was completely buried in the active site of amylase, forming H-bonds, van der Waals interactions and two π–π stacked interactions (Figure 3D). Likewise, 5-*O*-caffeoylquinic acid spanned the catalytic pocket of glucosidase via multiple H-bonds, a few van der Waals interactions, a hydrophobic and a π–π stacked interaction (Figure 3E). Together, these interactions are likely to account for the observed biological activity on the target enzymes.

Indeed, some previously published papers also showed that rutin, kaempferol and kaempferol 3-*O*-glucoside acted as cholinesterase inhibitors [41,42]. Concerning tyrosinase activity, numerous flavonoids, including rutin, kaempferol and kaempferol 3-*O*-glucoside and isorhamnetin, showed some inhibitory effects [43]. Rutin, kaempferol and its derivatives were also described as inhibitors of amylase and glucosidase, the enzymes targeted in diabetes type 2 therapy [44,45,46].

## 3. Materials and Methods

### 3.1. Plant Collection 

*Allium scorodoprasum* L. (Amaryllidaceae) flowers were collected during July 2021 on Kosmaj Mountain, Serbia. The flowers were lyophilized (LH Leybold, Lyovac GT2, Frenkendorf, Switzerland) and ground to a fine powder before further use. 

### 3.2. Extractions

Flower samples (2 g) were extracted overnight at 4 °C with 60 mL of each solvent listed in Table 1. The next day, samples were sonicated for 15 min and all were filtered through Whatman No. 4 paper. The plant residue was re-extracted using an additional 60 mL of the same solvent, at 4 °C, for 48 h, and the following procedure (sonication and filtration) was repeated. Extracts were evaporated at 40 °C on a rotary vacuum evaporator (Büchi R-210) to dryness.

### 3.3. UHPLC(−)HESI–QqQ-MS/MS Targeted Metabolomics Analysis

Quantification of the targeted compounds was performed using the Dionex Ultimate 3000 Ultra-High-Performance Liquid Chromatography (UHPLC) system (Thermo Fisher Scientific, Bremen, Germany) connected to a triple-quadrupole (QqQ) mass spectrometer (MS) (TSQ Quantum Access Max, Thermo Fisher Scientific, Basel, Switzerland).

A Syncronis C18 aqua analytical column (100 × 2.1 mm) with 1.7 μm particle size (Thermo Fisher Scientific, Fair Lawn, NJ, USA) was used for the chromatographic separation. The flow rate and composition of the mobile phases, as well as the gradient elution program, were previously described by Ivanov et al. [23]. The mass detector was equipped with a heated electrospray ionization (HESI) source operated in the negative ionization mode. The parameters of the HESI source and the other mass detector settings were previously described by Ivanov et al. [23]. The selected reaction monitoring (SRM) mode of the instrument was used for the quantification of the targeted compounds in the samples.

### 3.4. Antimicrobial Assays

#### 3.4.1. Isolation and Identification of Pathogenic Bacteria and Fungi Isolated from Infected Skin and Corneal Formations of Human Subjects

The samples containing bacteria, yeasts and dermatomycetes were collected at the Medical Military Academy (MMA), Belgrade, Serbia, from patients suffering from infections of the skin and/or corneal formations. A total of 19 human samples were collected. All of the patients were asked to provide informed consent to the use of their isolates in the experiments. The ethical committee of MMA approved the investigation (Approval No. 4/2021). 

The identification of microorganisms was performed on the Vitek MS system (bioMerieux SA, Marcy-l’Étoile, Lyon, France), which uses matrix-assisted laser desorption ionization time-of-flight (MALDI-TOF) to provide automated mass spectrometry microbial identification. Samples of bacteria, yeasts and dermatomycetes were prepared separately prior to analysis, according to the manufacturer’s instructions.

#### 3.4.2. Antibacterial and Anticandidal Activity

Strains used in the assay were skin isolates of bacteria, as well as the isolates of yeasts. The antibacterial and anticandidal activities of *A. scorodoprasum* extracts were evaluated by the microdilution method in 96-well microtiter plates. The minimum inhibitory concentrations (MIC) and minimal bactericidal/fungicidal concentrations (MBC/MFC) of extracts were determined as described previously [23]. Streptomycin and ketoconazole were used as positive controls.

#### 3.4.3. Antifungal Activity

The strains used in the assay were human isolates of dermatomycetes collected and identified as described in Section 3.4.1. The antifungal activity was evaluated by the modified serial dilution technique in 96-well microtiter plates. Spores of dermatomycetes were washed from 21-day-old cultures, using a sterile 0.85% physiological solution containing 0.1% Tween (*v*/*v*). The spore suspension was set with a sterile physiological solution to a concentration of 1.0 × 10^5^ per well. The plates containing extracts, spores and growth medium (Sabouraud Dextrose Broth (SDA)) were incubated at 37 °C for 72 h. 

The lowest concentrations without visible growth were defined as the MICs. MFCs were determined by the serial re-inoculation of 10 µL from the wells without growth into 100 µL of SDA, and re-incubated for 72 h at 37 °C. Ketoconazole was used as a positive control. 

#### 3.4.4. Activity of the Extracts against Biofilm Formation in *S. lugdunensis*

The impact of the selected extracts (EV7.5, EV5 and EV2.5) on *S. lugdunensis* biofilm formation was determined in 96-well microtiter plates with an adhesive bottom (Sarstedt, Germany). Tested extracts’ concentrations were MBC, MIC and MIC/2, while the bacterial inoculum was approximately 1 × 10^6^ CFU/well. After 24 h of incubation, each well was washed twice with PBS, biofilms were fixed with methanol and the plate was air-dried. The biofilm was stained with 0.1% crystal violet (Bio-Merieux, Craponne, France) for 30 min. Wells were washed with water and air-dried, while bound stain was dissolved in 100 μL of 96% ethanol (Zorka, Serbia). The absorbance was read at 620 nm on a Multiskan™ FC Microplate Photometer, Thermo Scientific™, and the percentage of inhibition of biofilm formation was calculated by the following formula:

[(A620 control − A620 sample)/A620 control] × 100.

#### 3.4.5. The Activity of Extracts against Formed Biofilms of *S. lugdunensis*

The assay was performed as described previously by Smiljković et al. [47]. *S. lugdunensis* (1 × 10^6^ CFU/well) was incubated in 96-well microtiter plates with an adhesive bottom (Sarstedt, Germany) in tryptic soy broth with 2% glucose at 37 °C, for 24 h, in order to establish biofilms. After incubation, wells were washed twice with sterile PBS and the remaining biofilm was treated with the 2xMBC, MBC and MIC of the extracts (EV7.5, EV5 and EV2.5) for an additional 24 h at 37 °C. Afterwards, the procedure was repeated as described in Section 3.4.4. 

### 3.5. Cytotoxicity towards HaCaT Cell Line

The cytotoxic effect of extracts was determined on a spontaneously immortalized keratinocyte cell line (HaCaT) using the crystal violet assay, as described previously [36], with some modifications. The extracts were dissolved in PBS to a final concentration of 8 mg/mL. HaCaT cells were grown in high-glucose Dulbecco’s Modified Eagle Medium (DMEM) supplemented with 10% fetal bovine serum (FBS), 2 mM L-glutamine and 1% antibiotic–antimycotic, at 37 °C, in a 5% CO_2_ incubator. Cells (10^4^ cells/well) were seeded in a 96-well microtiter plate with an adhesive bottom. After 48 h, the medium was removed and the cells were treated with various concentrations of the extracts in triplicate wells over 24 h. Afterwards, the medium was removed and the cells were washed twice with PBS and then stained with 0.4% crystal violet solution for 20 min. Crystal violet staining solution was removed, and cells were washed in a stream of tap water and left to air dry at room temperature. The absorbance of dye dissolved in methanol was measured at 570 nm (OD570) in a plate reader. The results were expressed as the IC50 value, indicating 50% of cell viability when compared with the untreated control. The solvent was used as a negative control. 

### 3.6. In Vitro Wound-Healing Assay on HaCaT Cell Line

The assay was performed as described in Ivanov et al. [23], with some modifications. HaCaT cells were grown until confluence was reached. The cell monolayer was scratched with a 200 μL tip. Floating cells were washed and cells were incubated in reduced DMEM supplemented with 1% FBS, 2 mM L-glutamine and 1% antibiotic–antimycotic (Invitrogen), containing 200 µg/mL of *A. scorodoprasum* flower extract. Cell migration was monitored with a Nikon Eclipse TS2 (Amsterdam, The Netherlands) 24 h after the wound was created and treated. The untreated control was used to measure wound closure under this condition, but without the addition of the extract. The results were presented as the percentage of wound closure during exposure to the tested extracts. 

### 3.7. Antioxidant Activity Assays

Antioxidant activities (DPPH and ABTS radical scavenging, reducing power (CUPRAC and FRAP), phosphomolybdenum and metal chelating (ferrozine method)) were determined using the methods previously described by Uysal et al. [48]. Details of the assays are provided in Supplementary Material S1.

### 3.8. Anti-Enzymatic Activities

All enzyme-inhibitory activities (cholinesterase (Elmann’s method), tyrosinase (dopachrome method), α-amylase (iodine/potassium iodide method) and α-glucosidase (chromogenic PNPG method)) were determined as previously described by Uysal at al. [48]. Details of the assays are provided in Supplementary Material S1.

### 3.9. Molecular Modeling

The following X-ray crystal structures of the target enzymes were retrieved from the Protein Data Bank (PDB) (https://www.rcsb.org/) (accessed on 6 October 2022): human AChE (PDB ID: 6O52) [49], human BChE (PDB ID: 6EQP) [50], human pancreatic alpha-amylase (PDB ID: 1B2Y) [51]. In the absence of the crystal structures of human tyrosinase and human glucosidase, *Priestia megaterium* tyrosinase (PDB ID: 6QXD) [52] and *Mus musculus* alpha-glucosidase (PDB ID: 7KBJ) [53] were also retrieved from the PDB and used to build their human models using the respective human sequences, UniProt IDs P14679 and P0DUB6. The detailed protocol of the homology modeling building was described previously [54]. To prepare proteins for docking, the pKa of the titratable residues in each protein was predicted using the “Playmolecule Protein Prepare” module [55], which was then used to prepare the proteins at a physiological pH of 7.4. The ligand 3D structures were retrieved from the PubChem database (https://pubchem.ncbi.nlm.nih.gov/) (accessed on 6 October 2022) and optimized using Frog2. Docking grid files were generated based on the size of each enzyme’s active site and the binding (x,y,z) coordinates of the cocrystal ligand in each crystal complex using AutoDockTools 1.5.6, and docking was performed using AutoDock 4.2.6 [56]. The docking protocol applied has been described previously [57]. The binding energy (docking) score of each ligand pose was calculated, and interactions with the enzymes were examined and visualized using Biovia DS Visualizer (Dassault Systèmes Biovia Software Inc., San Diego, CA, USA, 2012).

## 4. Conclusions

This is a pioneering study that offers scientific insights about the use of some natural pharmaceuticals empirically established in the medieval age. To the best of the authors’ knowledge, this is the first study investigating the chemical composition and different biological activities of wild-growing *A. scorodoprasum* flowers as influenced by different extraction solvent systems. Phenolic acids, flavonoid aglycones and glycosides were found in the investigated extracts; flavonoids dominated over phenolic acids. The B10 extract, with the lowest MIC of 0.125 mg/mL against *P. vulgaris,* proved to be the extract with the strongest antibacterial effect. The EW5 extract could be highlighted as the most promising antifungal agent with the best anticandidal effect (the lowest MIC was 0.06 mg/mL against the two yeasts *C. albicans* and *C. tropicalis*) and the strongest effect (MIC range 0.25–1 mg/mL) against almost all tested dermatomycetes. The extract with the strongest inhibition of biofilm formation in *S. lugdunensis* was also EW5, with 70.59%. The primary laboratory screening of cytotoxicity performed in this study led to the conclusion that *A. scorodoprasum* extracts are potentially safe for further applications. The extract that proved to be the best in the wound-healing process was EW5, with 100% healing capacity after 48 h of exposure in the HaCaT cell line. The highest antioxidant capacity was shown for extract EW5 in almost all of the assays. The most potent of all extracts against AChE and BChE was the E10 extract. The strongest effect on the enzyme tyrosinase was achieved by extract B10. We can conclude that the most potent extract for amylase and glucosidase inhibition was the B10 extract. Finally, molecular docking confirmed that the major tested phenolics were involved in the observed anti-enzymatic activity. Taken together, this study provides a profound basis for future in vivo investigations of *A. scorodoprasum* flower extracts and possible clinical applications.

## Figures and Tables

**Table 1:** Solvent systems used for the extraction and extraction yields.

Sample Code	Extraction Solvent	Yield % (*v*/*v*)
E10	Ethanol, 100%	3.87
EW7.5	Ethanol:Water, 75%:25%	36.99
EW5	Ethanol:Water, 50%:50%	6.63
EW2.5	Ethanol:Water, 25%:75%	11.42
W10	Water, 100%	9.38
B10	Butanol, 100%	7.03
BE7.5	Butanol:Ethanol, 75%:25%	13.21
BE5	Butanol:Ethanol, 50%:50%	9.05
BE2.5	Butanol:Ethanol, 25%:75%	11.76

**Table 2:** Phenolic constituents of the *Allium scorodoprasum* flower extracts in mg/kg dw.

Identified Compounds in mg/kg dw	E10	EW7.5	EW5	EW2.5	W10	B10	BE7.5	BE5	BE2.5
**3-*O*-Caffeoylquinic acid**	6.29 ± 0.34	3.41 ± 0.20	12.20 ± 1.63	40.90 ± 0.81	3.98 ± 0.55	17.38 ± 1.11	1.64 ± 0.13	3.90 ± 0.11	2.32 ± 0.30
**5-*O*-Caffeoylquinic acid**	173.75 ± 9.90	54.47 ± 0.82	211.36 ± 5.57	225.16 ± 1.73	19.93 ± 0.70	541.11 ± 13.11	10.35 ± 0.15	83.30 ± 1.00	51.62 ± 1.55
**Caffeic acid**	303.76 ± 6.99	80.10 ± 1.29	219.73 ± 0.78	82.45 ± 1.84	65.01 ± 2.10	154.46 ± 5.28	61.82 ± 1.66	80.87 ± 4.80	58.02 ± 2.62
**Isoorientin**	NF	NF	NF	128.19 ± 3.06	NF	NF	NF	NF	NF
**Rutin**	**2504.35 ± 68.46**	**1309.00 ± 22.86**	**5921.72 ± 202.28**	1117.89 ± 13.15	70.33 ± 1.80	1936.22 ± 47.94	140.85 ± 3.54	**631.88 ± 5.25**	477.32 ± 15.99
**Vitexin**	1.64 ± 0.13	0.44 ± 0.01	1.81 ± 0.25	24.18 ± 0.30	1.56 ± 0.16	1.76 ± 0.22	0.14 ± 0.01	0.44 ± 0.04	0.38 ± 0.07
**p-Coumaric acid**	215.96 ± 9.08	65.57 ± 0.76	192.52 ± 4.00	195.01 ± 3.31	180.16 ± 3.19	136.38 ± 4.16	14.67 ± 1.09	47.65 ± 2.53	43.39 ± 3.22
**Quercetin 3-*O*-glucoside**	136.42 ± 3.81	61.30 ± 0.81	347.91 ± 9.79	407.02 ± 1.29	17.26 ± 0.62	92.18 ± 3.88	5.49 ± 0.05	31.15 ± 1.26	26.46 ± 1.04
**Isorhamnetin 3-*O*-glucoside**	470.39 ± 0.19	191.56 ± 5.14	1673.95 ± 42.08	748.56 ± 16.46	NF	395.08 ± 17.90	34.78 ± 1.84	102.48 ± 2.68	133.64 ± 0.39
**Kaempferol 3-*O*-glucoside**	1774.76 ± 64.90	316.30 ± 3.54	1728.21 ± 11.31	**1362.05 ± 30.59**	27.60 ± 0.91	**2348.39 ± 69.81**	164.15 ± 5.49	526.13 ± 8.79	**503.54 ± 7.91**
**Eriodictyol**	NF	1.96 ± 0.03	7.27 ± 0.21	NF	NF	NF	NF	NF	NF
**Luteolin**	47.87 ± 3.46	7.94 ± 0.13	37.99 ± 1.95	105.17 ± 4.22	10.75 ± 0.42	23.85 ± 0.27	5.47 ± 0.20	11.00 ± 0.83	8.91 ± 0.92
**Quercetin**	21.52 ± 1.21	13.72 ± 0.16	50.85 ± 0.95	104.76 ± 4.17	180.37 ± 4.09	14.94 ± 0.89	1.73 ± 0.08	5.33 ± 0.04	2.92 ± 0.23
**Naringenin**	7.38 ± 0.51	1.53 ± 0.08	3.84 ± 0.22	1.51 ± 0.17	1.80 ± 0.14	4.86 ± 0.36	0.90 ± 0.10	2.25 ± 0.08	1.53 ± 0.17
**Apigenin**	NF	NF	NF	38.56 ± 2.14	129.01 ± 5.96	NF	NF	NF	NF
**Kaempferol**	1583.28 ± 111.10	703.46 ± 26.91	1427.17 ± 39.11	1121.81 ± 8.67	305.73 ± 11.30	1407.73 ± 58.77	**278.41 ± 3.20**	592.23 ± 21.26	383.78 ± 11.78
**Hispidulin**	96.59 ± 1.19	20.67 ± 1.59	58.93 ± 2.34	24.54 ± 0.28	30.78 ± 0.22	47.53 ± 1.94	16.79 ± 0.29	24.13 ± 0.31	17.34 ± 0.07
**Isorhamnetin**	266.40 ± 17.95	169.07 ± 8.97	565.76 ± 19.97	1201.72 ± 40.13	**605.67 ± 14.12**	282.41 ± 18.20	33.48 ± 1.44	110.04 ± 4.43	83.52 ± 2.14
**SUM**	7610.35	3000.50	12461.22	6929.48	1649.93	7404.29	770.68	2252.77	1794.68

The values are expressed as mean ± SD in mg/kg of dw. NF—not found.

**Table 3:** Pathogenic isolates from human skin and/or corneal formations identified by MALDI-TOF/MS.

	Species	Number of Isolates	Confidence Value	Label/Labels
Bacteria	*Staphylococcus epidermidis*	1	99.9	B45
	*Staphylococcus lugdunensis*	1	99.9	B43
	*Proteus vulgaris*	1	99.9	B44
Yeast	*Candida albicans*	1	99.9	Y177
	*Candida krusei*	1	99.9	Y454
	*Candida tropicalis*	1	99.9	Y149
	*Candida kefyr*	1	99.9	Y289
Dermatomycetes	*Microsporum fulvum*	2	99.9	D89
				D351
	*Trichophyton violaceum*	1	99.9	D1182
	*Trichophyton mentagrophytes*	2	99.9	D448
				D465
	*Microsporum canis*	2	99.9	D277
				D371
	*Trichophyton rubrum*	2	99.9	D460
				D1026

**Table 4:** Antimicrobial activity of different *Allium scrorodoprasum* flower extracts in mg/mL.

Extract mg/mL		*Staphylococcus lugdunensis* (B43)	*Staphylococcus epidermidis* (B45)	*Proteus vulgaris* (B44)	*Candida albicans* (Y177)	*Candida krusei* (Y454)	*Candida tropicalis* (Y149)	*Candida* *kefyr* (Y289)
E10	MIC	1	0.5	2	0.06	1	1	1
	MBC/MFC	2	1	4	0.125	2	2	2
EW7.5	MIC	0.5	0.5	2	0.125	2	0.125	0.125
	MBC/MFC	1	1	4	0.25	4	0.25	0.25
EW5	MIC	0.5	0.5	1	0.06	1	0.06	0.5
	MBC/MFC	1	1	2	0.125	2	0.125	1
EW2.5	MIC	0.5	1	2	0.125	2	0.25	0.5
	MBC/MFC	1	2	4	0.25	4	0.5	1
W10	MIC	4	0.5	0.5	0.25	0.5	0.5	0.5
	MBC/MFC	8	1	1	0.5	1	1	1
B10	MIC	4	0.5	0.125	0.5	0.5	0.5	0.5
	MBC/MFC	8	1	0.25	1	1	1	1
BE7.5	MIC	1	0.5	1	0.5	0.5	0.5	0.5
	MBC/MFC	2	1	2	1	1	1	1
BE5	MIC	4	0.5	1	0.5	0.5	0.5	0.5
	MBC/MFC	8	1	2	1	1	1	1
BE2.5	MIC	4	0.5	4	0.5	4	4	4
	MBC/MFC	8	1	8	1	8	8	8
Streptomycin	MIC	0.003	0.1	0.003	-	-	-	-
	MBC	0.006	0.2	0.006	-	-	-	-
Ketoconazole	MIC	-	-	-	0.015	0.015	0.015	0.015
	MFC	-	-	-	0.030	0.030	0.030	0.030

**Table 5:** Antifungal activity of different *Allium scorodoprasum* flower extracts against isolated dermatomycetes in mg/mL.

Extract mg/mL		*Microsporum fulvum* (D89)	*Microsporum fulvum* (D351)	*Trichophyton violaceum* (D1182)	*Trichophyton mentagrophytes* (D448)	*Trichophyton mentagrophytes* (D465)	*Microsporum canis* (D277)	*Microsporum canis* (D371)	*Trichophyton rubrum* (D460)	*Trichophyton rubrum* (D1026)
E10	MIC	2	1	2	2	2	1	1	1	1
	MFC	4	2	4	4	4	2	2	2	2
EW7.5	MIC	2	2	2	2	2	1	1	1	1
	MFC	4	2	4	4	4	2	2	2	2
EW5	MIC	1	1	1	1	1	0.5	0.5	0.25	0.25
	MFC	2	2	2	2	2	1	1	0.5	0.5
EW2.5	MIC	1	2	2	1	1	0.5	0.5	0.25	0.25
	MFC	2	4	4	2	2	1	1	0.5	0.5
W10	MIC	4	4	4	4	4	2	2	2	2
	MFC	8	8	8	8	8	4	4	4	4
B10	MIC	4	4	4	4	4	4	4	4	4
	MFC	8	8	8	8	8	8	8	8	8
BE7.5	MIC	4	4	4	4	4	4	4	4	4
	MIC	8	8	8	8	8	8	8	8	8
BE5	MIC	4	4	4	4	4	4	4	4	4
	MFC	8	8	8	8	8	8	8	8	8
BE2.5	MIC	4	4	4	4	4	4	4	4	4
	MFC	8	8	8	8	8	8	8	8	8
Ketoconazole	MIC	0.025	0.025	0.0125	0.0125	0.0125	0.25	0.25	0.0125	0.0125
	MFC	0.050	0.050	0.0250	0.0250	0.0250	0.50	0.50	0.0250	0.0250

**Table 6:** Antibiofilm activity of different *Allium scrorodoprasum* flower extracts on biofilm formation of *Staphylococcus lugdunensis*.

Extract	Inhibition Percentage [%]		
	MIC/2	MIC	MBC
EW7.5	43.75%	65.44%	66.18%
EW5	46.32%	63.97%	70.59%
EW2.5	44.11%	51.84%	62.5%

**Table 7:** Antibiofilm activity of different *Allium scrorodoprasum* flower extracts on formed biofilm of *Staphylococcus lugdunensis*.

Extract	Inhibition Procentage [%]		
	MIC	MBC	2xMBC
EW7.5	NI	NI	NI
EW5	NI	NI	NI
EW2.5	NI	NI	46.6%

NI—no inhibition of formed biofilm.

**Table 8:** Effects of the *A. scorodoprasum* flower extracts on wound healing in HaCaT cells. Results are presented as percentage of wound closure. NE—no effect on migration.

Extract	Wound Closure (%)
E10	81.00 ± 0.97
EW7.5	17.34 ± 1.74
EW5	100.00 ± 0.00
EW2.5	23.18 ± 4.58
W10	23.16 ± 0.98
B10	26.33 ± 1.22
BE7.5	23.66 ± 2.05
BE5	13.17 ± 2.88
BE2.5	17.14 ± 1.11
Control	0.83 ± 0.08

**Table 9:** Antioxidant activity of different *Allium scrorodoprasum* flower extracts.

Extract	DPPH mg TE/g	ABTS mg TE/g	CUPRAC mg TE/g	FRAP mg TE/g	Metal Chelating mg EDTAE/g	Phosphomolybdenum mmol TE/g
Mean ± SD						
**E10**	16.03 ± 0.45	32.56 ± 0.70	55.35 ± 0.61	**49.34 ± 0.98**	7.69 ± 0.32	1.79 ± 0.01
**EW7.5**	16.33 ± 0.73	42.09 ± 0.53	50.86 ± 0.56	44.06 ± 0.04	16.64 ± 0.18	1.31 ± 0.05
**EW5**	**18.66 ± 0.49**	**44.04 ± 0.19**	**56.26 ± 1.37**	48.83 ± 0.29	**18.60 ± 0.55**	**1.83 ± 0.11**
**EW2.5**	15.50 ± 0.70	40.71 ± 1.30	45.27 ± 0.38	37.80 ± 0.64	16.33 ± 0.15	1.09 ± 0.10
**W10**	12.05 ± 0.01	34.76 ± 0.37	41.31 ± 0.85	39.78 ± 0.77	12.40 ± 0.29	0.99 ± 0.08
**B10**	12.33 ± 0.27	27.95 ± 0.37	48.17 ± 1.16	44.26 ± 0.89	8.67 ± 0.47	1.30 ± 0.04
**BE7.5**	10.92 ± 0.32	27.28 ± 0.52	43.07 ± 0.81	40.03 ± 0.54	6.11 ± 0.70	1.13 ± 0.08
**BE5**	2.50 ± 0.34	8.46 ± 0.86	20.94 ± 0.87	18.16 ± 0.17	16.76 ± 0.67	0.60 ± 0.02
**BE2.5**	3.37 ± 0.72	10.35 ± 1.42	22.36 ± 0.72	19.72 ± 0.26	11.12 ± 1.04	0.56 ± 0.02

Values are reported as mean ± SD of three parallel measurements. TE: Trolox equivalent; EDTAE: EDTA equivalent.

**Table 10:** Anti-enzymatic activity of *Allium scorodoprasum* flower extracts.

Extract	AChE mg GALAE/g	BChE mg GALAE/g	Tyrosinase mg KAE/g	Amylase mmol ACAE/g	Glucosidase mmol ACAE/g
	Mean ± SD na: not active				
**E10**	3.05 ± 0.03	3.50 ± 0.16	65.24 ± 3.20	**0.28 ± 0.01**	0.86 ± 0.02
**EW7.5**	3.06 ± 0.03	2.61 ± 0.14	53.24 ± 2.29	0.24 ± 0.00	**0.90 ± 0.01**
**EW5**	2.99 ± 0.07	1.81 ± 0.23	43.96 ± 5.15	0.20 ± 0.01	0.89 ± 0.01
**EW2.5**	3.04 ± 0.01	2.13 ± 0.19	53.57 ± 2.30	0.10 ± 0.01	**0.90 ± 0.00**
**W10**	2.98 ± 0.06	2.54 ± 0.19	41.23 ± 1.95	0.19 ± 0.00	na
**B10**	**3.08 ± 0.07**	3.42 ± 0.14	**75.66 ± 4.83**	**0.28 ± 0.01**	**0.90 ± 0.00**
**BE7.5**	2.83 ± 0.00	3.34 ± 0.02	65.34 ± 8.12	0.20 ± 0.15	0.89 ± 0.00
**BE5**	na	3.96 ± 0.06	50.74 ± 6.33	0.25 ± 0.01	0.88 ± 0.00
**BE2.5**	na	**3.99 ± 0.08**	52.77 ± 3.85	0.23 ± 0.01	0.88 ± 0.01

Values are reported as mean ± SD of three parallel measurements. GALAE: Galantamine equivalent; KAE: Kojic acid equivalent; ACAE: Acarbose equivalent.

## Data Availability

Data is available within article and supplementary materials.

## Supplementary Materials

The following supporting information can be downloaded at: https://www.mdpi.com/article/10.3390/ph16010021/s1, Details of the antioxidant and anti-enzymatic assays.
